# An Ishmaelite

**Published:** 1888-09-29

**Authors:** Leslie Keith

**Affiliations:** Author of "The Chilcotes," "Uncle Bob's Niece," "East and West," etc.


### AN ISHMAELITE.

By LESLIE KEITH,

Author of "The Chilcotes," "Uncle Bob's Niece," "East and West," etc.

(Concluded from p. 410.)

### CHAPTER XXVI.—THE LOVE THAT SAVED.

FRED lingered for a long time in the valley of shadows, and only slowly, very slowly, climbed up the steep road that led back to health.

On the day when his recovery was pronounced certain, Arthur asked Delia to go out with him. It was June now—the time of high summer, the month of roses. No roses bloomed on the crowded Bermondsey streets, but there was content enough in their two hearts to transfigure the sordid City ways, and turn the hoary old world into a paradise.

They strayed into the churchyard where Fred and he had once sat and talked; and they rested on the same grey tombstone—the old church almost hidden from their view by the foliage of the trees, green for a brief summer. And now that they were together and alone, there seemed to be nothing to explain; they were content for a little while to be silent, in that blissful mutual consciousness of each other's love that made words superfluous. She let her hand rest in Arthur's when he took it in both his own.

"Delia," he said at last, "you know that I love you, and have longed for you these many months, and now—I think you have given yourself to me—but will you say it just once? say, "Arthur, I will try to think you worth loving a little."

She looked up at him with blushes on her cheeks and a light in her shy, soft eyes.

"Arthur," she murmured, "I—I love you."

They sat looking at each other in that young wonder that each new pair experiences over again; to be near each other and to care for each other was enough, and by-and-by, when Arthur spoke out of the fulness of his heart, it was but to tell the old story over again.

"I have something to tell you," said Delia, after a while, "that was why I came with you here—where it is quiet."

"What have you to tell me, Delia?"

"It is something about the future."

"Our future is for you to arrange," said Arthur, gently. "When I asked for you I promised your father that you should never be taken away from him or your mother and sister, where you could not see them or be with them as often as you willed; every day if you feel the wish. You shall choose where we shall live and how."

"Ah," she said, and the tears rose, "but they are going where I shall not see them every day. My uncle out in Australia is dead. That is my news. We heard of it only yesterday. He has left some of his money to my father and mother, and some of it—most of it, I think—to me. He remembered me when I was little."

"So you are an heiress, are you, Delia? I am sorry for it. I wish he had left his money to a hospital; it would have been such a dear pleasure to give you everything."

"Oh, you will give me everything still," she said, simply, "But I am a little glad of the money, though I wish he had not died, because it makes us less unequal. It is not for myself," she went on timidly, seeing the protest in his face, "I think I could be content to take everything from you, but your friends—they will not think it so strange, and my father will consent now."

"He couldn't have held out," said Arthur, strongly.

"Yes," she said; "you don't know how he can hold out where he thinks it is a duty."

"Oh, don't I! I have fought with him every day for six months, Delia!"

"At least, even if he had consented, it would have vexed him," she went on, "but Uncle Peter's money has reconciled him."

"Oh, the pride of him!" laughed Arthur. "As if my friends would not love my Delia for what she is in herself!"

But for all that, the canny Scot was right. Arthur's marriage would no longer be criminal in the eyes of the world where, after all, he had to live, but only eccentric and romantic and interesting, and society would give this little sleeping beauty new wakened to love and wealth a very kind welcome.

"Father and mother and Aunt Betsy are going to the home he has made out there, and Barbara——"

"And Fred," said Arthur sadly. "It was I who was to have gone with him; but Barbara will do for him far more than ever I could do. I think poor Aunt Helen, if she could know, would be happy about him at last."

"Perhaps she does know," said Delia softly.

"I used to think that he would share our home; but it is better as it is."

"And we shall lose them all," said Delia mournfully; "and the old home gone too."

"And the poor library done for! Delia, shall we give Gillespie Street a new library as our wedding gift?"

But she did not answer. She was busy with a thought of her own that seemed to trouble her.

"Arthur," she said beseechingly, "you—you would never have been ashamed of them if they had lived on here—here in this poor place, and had not gone away where we shall not see them—my father a shop-keeper?"

Then, seeing the wounded love and indignation in his face, she took his hand and bent her head over it.

"Oh," she said, "it was cruel, wicked of me to ask. You never were ashamed of us, else why did you stoop to me? You came to us. You did not forsake your cousin, though I think, perhaps, there was some sadness in his life that sent him here to live so differently. Will you forgive me?

"How shall I prove my sincerity, Delia? Shall we ask your father and mother to give up this new plan and to remain here with us? or shall we cast in our lot with them and cross the seas too? It is for you, only for you, my dear, to choose."

"No," she said, "there is your uncle; you must think of him. I saw him when he came to see Fred. He is old and sad and lonely; you cannot leave him."

"Then you must be a daughter to him, Del, poor old man, and I will try—I'm afraid I have not always tried—to be what Fred might have been to him."

Somewhat on these lines the future of those with whom we have journeyed so far arranged itself in course of time, though it was late in the year before that exodus of which Delia and Arthur talked in the high summer took place.

When Fred won that hard fight with death and came out of the struggle faint and bruised, but with a clutch on life once more, it was to find Barbara by his side, her face bent over his and stirred with many emotions.

"Barbara," he said faintly, "where am I?"

"You have been ill," she said, "but you are going to live."

"I remember now, it was the fire"—

"Yes," she controlled herself, "do not think of it yet. You are going to get well and strong. You are given back to us for whom you gave yourself."

Not all at once did he realise what this coming back meant, what of hope and undreamed of bliss it held. He struggled against the temptation to yield; he strove to curb the yearning for those joys that other men might claim and wear proudly.

"It is too great a sacrifice, I dare not accept it," he said. "My own people cast me out to wander like Ishmael in the wilderness, and you would take me in and give me this great boon. I cannot take it, Barbara."

Barbara looked at him with a deepening of her smile. She tried to speak lightly, though her lips were tremulous.

"You are no longer your own," she said. "You gave me my life, and now you owe yours to me. Ask the doctor he will tell you what a splendid nurse I made. He thinks nursing is my vocation. Perhaps it is; shall I don the livery, and hide myself behind hospital walls, Fred?"

"You would shine there or anywhere," he said sadly, looking at the girl's broad brow and calm, strong face. "You are made for high things, Barbara."

But Barbara had no desire to shine; she grew cold before the image of a life that had renounced the highest human blessedness for the poor applause of the cultured few; for even in these days of an emancipated womanhood there are hearts to whom love is more sweet than praise. Barbara's needs went out in the longing for some one to care for, to shield, to protect; upon whom to spend the wealth of a very generous affection, and who shall blame Fred if, in the end, he made surrender, and humbly took this great boon?

Possibly he got more than he deserved, but there have been instances in the world's history before of men who have married wives a great deal better than they, and it is at least certain that Barbara never suffered a regret over the choice she voluntarily made.

To have set out on the hard and arid path of renunciation, the sad unfriended road of penance where the sun never shine nor flowers star the wayside, and then to have heaven open and this glimpse of bliss revealed to him! Barbara came to him as an angel, to loose his bonds, to lead him out of the depths to those purer heights where she dwelt; he was awe-stricken before her goodness, but he put his hand in hers and thanked God for the great love that stooped to save him.

He was much moved when he heard of that visit his father had paid to his sick-bed while he lay scarcely conscious of what was passing around him.

"I have been a bad son to him," he said. "I should like to ask his forgiveness before we go. Ah, Barbara, suppose I wound your hopes of me too—suppose you should ever repent that you have stooped to me?"

"Hush," she said tenderly, "do not speak of me as if I had not been in the battle too; if I have been saved from some sins it is because my temptations have been less. We will begin the world anew; we will help each other."

So she was ever ready to encourage him, to cheer, to strengthen him, to be patient with him in his darker moods, to love him always.

Did he rise in the future to all that we could wish Barbara's husband to be?

When we think how often the bravest succumbs, how often high endeavour falls short, how stained is the brightest record, we must not look for any miracle.

Perhaps to the end of his life Fred remained weak, and never became a hero, even to those who loved him most, but in one thing he never failed—in his love and reverence for Barbara. To have wounded or disappointed her, to have cast even a shadow over her happiness, he would have held the deepest disgrace of a life that had known many lapses, and his best hope of growing to be worthy of her was his daily prayer that he might become so.

THE END.

